# Evaluating the Associations between Dietary Vitamin Intake and Atopic Dermatitis: A Regional Cross-Sequential Study among Singapore and Malaysia Young Chinese Adults

**DOI:** 10.1016/j.xjidi.2025.100387

**Published:** 2025-05-27

**Authors:** Jun Jie Lim, Kavita Reginald, Yee-How Say, Mei Hui Liu, Fook Tim Chew

**Affiliations:** 1 Department of Biological Sciences, Faculty of Science, National University of Singapore, Singapore, Singapore; 2 Department of Biological Sciences, School of Medicine and Life Sciences, Sunway University, Selangor, Malaysia; 3 Department of Biomedical Science, Faculty of Science, Universiti Tunku Abdul Rahman (UTAR), Perak, Malaysia; 4 Department of Food Science & Technology, Faculty of Science, National University of Singapore, Singapore, Singapore

**Keywords:** Atopic dermatitis, Eczema, Epidemiology, Inflammatory skin diseases, Public health research

## Abstract

This study demonstrates that naturally derived vitamins, estimated from whole foods in the diets of young Chinese adults from Singapore and Malaysia, are associated with atopic dermatitis (AD). Higher intake of vitamins E, K1, C, B2, and D was associated with lower odds of AD. The protective effect of vitamin C was not confounded by smoking and enhanced by higher fruit intake. These findings underscore the potential role of dietary vitamins in mitigating AD risk and support further research into whole-food-based dietary strategies for AD management.

## Introduction

Atopic dermatitis (AD) is a prevalent chronic inflammatory skin condition affecting millions worldwide, including young adults, and significantly impairing quality of life through physical discomfort and psychological distress (Lim et al, 2022). Although research on dietary factors in AD has focused on vitamin supplementation in pediatric populations, the role of naturally derived vitamins from whole foods in the adult diet remains poorly understood. This cross-sequential study aims to explore the association between AD and dietary vitamin intake using amount-based dietary indices in young adults from Singapore and Malaysia, with the objective of informing evidence-based dietary strategies for AD management.

## Results and Discussion

**Figure 1:**
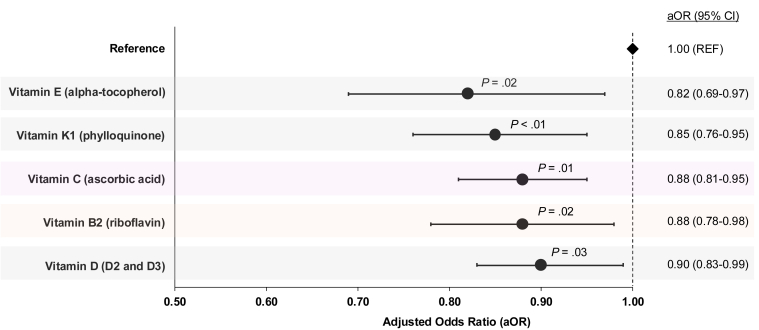
**OR plot depicting the association between dietary vitamin intake and atopic dermatitis among young Chinese adults from Singapore and Malaysia.** Statistical significance is indicated as *P* < .05, with *P*-values adjusted for multiple comparisons using the false discovery rate. Multivariable logistic regression models were adjusted for age (years), sex, body mass index (Asian classification), parental atopic diseases, alcohol intake, income categories, and physical activity. For fat-soluble vitamins (D, E, and K1), total fat intake (g/serving/week) was additionally controlled for. Smoking was adjusted for in the analyses of vitamin C, whereas total energy intake (kcal/serving/week) was adjusted for in the analysis of vitamin B2. The dotted vertical line represents the reference line at aOR of 1.00. aOR, adjusted OR; CI, confidence interval; REF, reference.

Figure 1 shows the adjusted OR (aOR) comparing the association between dietary vitamin intakes and AD. Initially, high vitamin E intake was associated with higher odds of AD. However, after adjusting for total fat intake, the association for vitamin E shifted to a protective effect (aOR = 0.82, 95% confidence interval [CI] = 0.69–0.97, *P* = .02), highlighting the confounding effect of fat intake. This adjustment suggests that the true association between vitamin E and AD is protective, likely due to its role as a potent antioxidant that mitigates oxidative stress and inflammation associated with AD. Supplementation studies have also supported these findings, demonstrating improvements in AD clinical symptoms with doses ranging from 400 to 1200 IU/day over 4–8 months. The use of topical vitamin E was not specifically captured in these studies, but previous research suggests that dietary supplementation of vitamin E alone may have beneficial effects on AD symptoms (Teo et al, 2021).

Our results showed a modest dose–response relationship between higher vitamin D intake and lower odds of AD after adjusting for fat intake (aOR = 0.90, 95% CI = 0.83–0.99, *P* = .03). Although these findings suggest a potential protective role of dietary vitamin D, caution is warranted owing to the complexity of vitamin D metabolism, conflicting evidence in the literature (Mesquita et al, 2013), and the potential confounding effect of sun exposure (Wacker and Holick, 2013). However, in a subset analysis (n = 1016), we observed that the impact of sun exposure was minimal, and the protective association between dietary vitamin D intake and AD remained significant (aOR = 0.69, 95% CI = 0.48–0.99, *P* < .05). Although several intervention studies have examined vitamin D in relation to AD, longitudinal studies are needed to track the effects of vitamin D from whole foods, supplements, and sun exposure to better understand its role in the development and management of AD.

Vitamins K1 (aOR = 0.85, 95% CI = 0.76–0.95, *P* < .001) and B2 (aOR = 0.88, 95% CI = 0.78–0.98, *P* = .02) showed a protective dose–response relationship with lowered odds of AD. Dietary vitamin C intake also significantly lowered the odds of AD even after adjusting for smoking (aOR = 0.88, 95% CI = 0.81–0.95, *P* = .01). However, this association was lost after adjusting for fruit intake, suggesting that the effect might primarily stem from vitamin C in fruit. The stratified analysis further revealed that low-moderate vitamin C intake with high fruit intake was sufficient to lower AD odds (aOR = 0.64, 95% CI = 0.46–0.88, *P* = .01). These findings highlight the potential of nutrient-dense dietary patterns, particularly those high in fruit and vegetables, as a practical approach to supporting long-term management of AD.

No statistically significant associations were found between dietary intake of vitamins A, B1, B3, B5, and AD. Furthermore, dietary vitamin intake did not show any significant associations or interactions with symptom recovery among individuals with AD. These results suggest that although certain vitamin intake may influence AD development, it does not appear to affect symptom recovery. Overall, the findings emphasize the complex interplay between nutritional factors and AD pathophysiology.

This study has several limitations. The short food frequency questionnaire, although validated, may not fully capture long-term dietary patterns or accurately assess the intake of multiple vitamins simultaneously. Analyzing data at the food level rather than the nutrient level introduces variability because nutrient content can differ significantly within food groups (eg, refined vs whole grains). Duration of food intake may impact the bioavailability of vitamins. The lack of dietary supplement data limits our ability to differentiate between natural dietary intake and supplementation, potentially influencing the observed associations.

Nonetheless, the study’s strengths include its large, diverse cohort; the use of validated food frequency questionnaires and robust diagnostic criteria; and its focus on natural dietary patterns, offering real-world insights into the role of dietary vitamins in AD from an Asian regional perspective. Incorporating biomarkers, such as serum levels of specific vitamins (eg, C and D), would offer more accurate measures of actual vitamin intake, clarifying the relationship between vitamins and AD by reflecting bioavailability (Zheng et al, 2023). Furthermore, future randomized controlled trials are needed to establish optimal vitamin levels, intake duration, and their long-term effects on AD progression and symptom management.

## Materials and Methods

Between 2005 and 2022, 13,561 participants were voluntarily recruited for the Singapore/Malaysia Cross-sectional Genetics Epidemiology Study. This study was conducted in adherence to the Declaration of Helsinki and Good Clinical Practices and in compliance with local regulations. Ethical approval was granted by National University of Singapore Institutional Review Board (reference codes NUS-07-023, NUS-09-256, NUS-10-445, NUS-13-075, NUS-14-150, and NUS-18-036) and ethics committees from Universiti Tunku Abdul Rahman (reference U/SERC/03/2016) and Sunway University (reference SUREC 2019/029). Participants included university students, staff, and the public from Singapore and Malaysia, randomly selected to minimize selection bias. Written, informed consent was obtained from participants in compliance with ethical guidelines. No specific inclusion or exclusion criteria were applied. This study focused on participants of Chinese ethnicity, given their majority representation.

Given the central role of allergic sensitization in AD, we defined AD as the presence of both sensitization and recent symptoms. Sensitization was assessed by a positive skin prick test response to common house dust mite allergens. Among those sensitized, AD was defined by the presence of recurrent flexural rash in the past 12 months (n = 1979, 14.6%) on the basis of the UK Working Party’s (Williams et al, 1994) and Hanifin and Rajka diagnostic criteria (Hanifin and Rajka, 1980). All clinical assessments, including evaluation of other skin symptoms related to AD, were conducted by trained personnel and verified by a dermatologist. Nonallergic, noneczema participants (n = 3650) served as the reference group (Table 1). Dietary intake was assessed using a validated, investigator-administered semiquantitative food frequency questionnaire covering 16 common food groups (Ellwood et al, 2013). Dietary vitamin indices were developed on the basis of United States Department of Agriculture nutritional data and participant-reported intake frequencies, focusing on key vitamins with established relevance to skin and immune health (Lim et al, 2025c). Intake scores were calculated and are presented in Table 2, whereas their associations with AD are presented in Figure 1. The skin prick test protocol and dietary index scoring were consistent as previously described (Lim et al, 2025a, 2025b, 2025c, 2024a, 2024b, 2023a, 2023b, 2023c). Logistic regression models, adjusting for covariates such as age, sex, body mass index, parental atopic disease, lifestyle, and income categories, assessed the associations between dietary vitamin intake and AD. Statistical analyses, including synergy factor assessments (Cortina-Borja et al, 2009), were conducted, with false discovery rate adjustments (Benjamini and Hochberg, 1995) applied to ensure robust interpretation of results. Further information on methods and findings is available in the repository (https://osf.io/f7mpq/?view_only=af2d76f70ddb41238f957727ce27422f).

**Table 1:** Demographics of the Young Chinese Adults from the 13,561 SMCGES Cohort

Variables	Nonallergic Noneczema (n = 3650)	Atopic Dermatitis^1^ (n = 1979)	*P*-Value
Mean age ± SD	22.73 ± 6.45	22.45 ± 6.04	1.10 × 10^−1^
Sex			
Male	1068 (29.3)	804 (40.6)	**<2.20** × **10^−16^**
Female	2582 (70.7)	1175 (59.4)	
BMI, Asian class (kg/m^2^)			
Mean BMI ± SD	20.76 ± 3.12	21.20 ± 3.43	**8.50** × **10^−6^**
Healthy (18.0–23.0)	2060 (56.4)	1020 (51.5)	**9.67** × **10^−5^**
Underweight (<18.0)	697 (19.1)	336 (17.0)	
Overweight (>23.0)	519 (14.2)	354 (17.9)	
NA	374 (10.2)	269 (13.6)	
Parental atopic diseases^2^			
None	3050 (83.6)	1313 (66.3)	**<2.20** × **10^−16^**
At least 1	518 (14.2)	638 (32.2)	
NA	82 (2.25)	28 (1.41)	—
Alcohol consumption			
Nondrinker	1731 (47.4)	822 (41.5)	**3.99** × **10^−4^**
Occasional drinker	1684 (46.1)	1003 (50.7)	
Frequent drinker	65 (1.8)	40 (2.0)	
NA	170 (4.7)	114 (5.8)	—
Income category per household per capita (SGD)			
<$2000	885 (24.2)	316 (16.0)	**1.05** × **10^−10^**
$2000–3999	1126 (30.8)	610 (30.8)	
$4000–5999	689 (18.9)	443 (22.4)	
>$6000	679 (18.6)	492 (24.9)	
NA	271 (7.42)	118 (5.96)	—
Engagement in physical activities			
Never or only occasional	1418 (38.8)	662 (33.5)	**5.37** × **10^−6^**
Once or twice per week	1841 (50.4)	1037 (52.4)	
Most or all days	365 (10.0)	265 (13.4)	
NA	26 (0.71)	15 (0.76)	—

Abbreviations: BMI, body mass index; NA, not applicable; SGD, Singapore dollar; SMCGES, Singapore/Malaysia Cross-sectional Genetics Epidemiology Study.

A student *t*-test was used to analyze the difference in mean age and mean BMI, whereas a chi-square test was used to examine the difference in other categorical variables (sex, BMI, alcohol consumption, income categories, and engagement in physical activities). *P*-values < .05 were considered statistically significant and were written in bold.

1 Atopic dermatitis is defined by a positive skin prick test to *Blomia tropicalis* and/or *Dermatophagoides pteronyssinus* and recurrent itchy flexural rash within the past 12 months.

2 Parental atopic diseases refer to a diagnosis of allergic rhinitis, atopic dermatitis, or allergic asthma in either biological parent.

**Table 2:** Descriptive Statistics for Estimated Weekly Intake per Serving of Key Fat-Soluble and Water-Soluble Vitamins among Participants with Atopic Dermatitis and Nonallergic Noneczema Controls in the SMCGES Cohort

Dietary Indices	Nonallergic, Noneczema (n = 3650)						Atopic Dermatitis (n = 1979)					
	Mean	SD	Min	Max	33^rd^ Percentile	66^th^ Percentile	Mean	SD	Min	Max	33^rd^ Percentile	66^th^ Percentile
Estimated intake per wk per serving for fat-soluble vitamins												
Vitamin A (μg)	4064.0	3162.0	0.0	14,889.0	2160.0	4599.0	4510.0	3493.0	0.0	14,889.0	2475.0	5041.0
Vitamin D (mg)	25.2	10.7	0.0	43.4	21.0	29.3	25.2	10.6	0.0	43.4	21.1	29.2
Vitamin E (mg)	55.5	40.6	0.0	219.3	32.9	64.1	50.3	45.3	0.0	219.3	35.5	67.0
Vitamin K1 (μg)	969.7	306.8	0.0	1803.6	931.1	1083.5	969.9	306.8	0.0	1803.6	930.0	1096.8
Estimated intake per wk per serving for water-soluble vitamins												
Vitamin B1 (mg)	7.0	3.0	0.0	19.7	5.6	7.6	7.4	3.3	0.0	19.7	5.8	8.0
Vitamin B2 (mg)	9.7	3.2	0.0	18.8	8.2	11.0	9.9	3.3	0.0	18.8	8.3	11.2
Vitamin B3 (mg)	102.4	31.9	0.0	195.7	86.5	116.7	104.1	32.7	0.0	195.7	87.7	118.8
Vitamin B5 (mg)	27.7	8.5	0.0	52.2	23.4	31.6	29.3	8.7	0.0	52.2	23.8	32.2
Vitamin C (mg)	285.2	96.0	0.0	458.0	222.9	344.9	276.7	101.1	0.0	458.0	213.0	343.1

Abbreviations: Max, maximum; Min, minimum; SMCGES, Singapore/Malaysia Cross-sectional Genetics Epidemiology Study.

## Ethics Statement

The study adhered to the ethical standards of the Declaration of Helsinki and Good Clinical Practices, with approvals from the National University of Singapore Institutional Review Board (NUS-07-023, NUS-09-256, NUS-10-445, NUS-13-075, NUS-14-150, and NUS-18-036) and the ethical committees at Universiti Tunku Abdul Rahman (reference code U/SERC/03/2016) and Sunway University (reference code SUREC 2019/029) in Malaysia. Written, informed consent was obtained from participants in compliance with ethical guidelines.

## Data Availability Statement

The data underlying this article will be shared on reasonable request to the corresponding author (FTC). Further information on methods and findings is available in the repository (https://osf.io/f7mpq/?view_only=af2d76f70ddb41238f957727ce27422f).

## ORCIDs

Jun Jie Lim: http://orcid.org/0000-0001-5485-1749

Kavita Reginald: http://orcid.org/0000-0003-1530-5934

Yee-How Say: http://orcid.org/0000-0003-2363-5239

Mei Hui Liu: http://orcid.org/0000-0001-7405-7008

Fook Tim Chew: http://orcid.org/0000-0003-1337-5146

## Conflict of Interest

FTC reports grants from the National University of Singapore, Singapore Ministry of Education Academic Research Fund, Singapore Immunology Network, National Medical Research Council (Singapore), Biomedical Research Council (Singapore), National Research Foundation (Singapore), Singapore Food Agency, Singapore’s Economic Development Board, and the Agency for Science Technology and Research (Singapore), during the conduct of the study, and consulting fees from Sime Darby Technology Centre, First Resources, Genting Plantation, Olam International, Musim Mas, and Syngenta Crop Protection, outside the submitted work. The remaining authors state no conflict of interest.
